# Therapeutic Pearls at Random Strung

**Published:** 1881-02

**Authors:** 


### THERAPEUTIC PEARLS AT RANDOM STRUNG.

### Burns and Scalds.

℞ Beeswax melted and strained, - - f. ℥.

Flaxseed or Olive Oil, - - - - f ℥ ij.

Tannic Acid. - - - - - - f ʒ i.

Mix as follows: Put in a clean tin vessel and mix the wax; add the oil and stir until they are thoroughly incorporated with each other, then sit off the fire and continue to stir at short intervals till cold. Add the tannic acid immediately after sitting off, and add 6 or 8 drops of fluid carbolic acid to the ounce.

Spread on strips of old cloth and apply.

### Bicarbonate Soda in Burns.

A saturated solution has been highly recommended in scalds and burns. It is applied by saturating cloths in the solution and keeping them constantly wet over the burned surface.

### Dr. Bartholow's Prescription for Asthma.

℞ Potass. iod. - - - - - - gr. xv.
Potass. bromid., - - - - - gr. xx.

M. S. Take every four or six hours.—*Chicago Med. Review.*

### Surgical Treatment of Epistaxis.

Dr. Thurston depends upon the well-known fact that liquid injected into one nostril returns by the other, and in cases of epistaxis introduces the nozzle of a syringe into the nostril not bleeding, and holds it firmly. A stream of cold water thrown in thus, washes out all the clots from the bleeding nostril, and often arrests the bleeding. If not efficient for this purpose, he uses a dilute solution of perchloride of iron.—*Brit. Medical Journal.*

### For Burns.

℞ Carbolic acid, part j.; balsam fir, oleum olivæ, pure, *aa* parts xvi.

### Chapping of the Skin.

| | | |
|---|---|---|
| ℞ | Oxide Zinc. - - - - - - | gr. xx. |
| | Tannic acid, - - - - - - | gr. v. |
| | Glycerine, - - - .. - | ℥ ix. |
| | Tinct. Benzoin, - - - - | ℥ ss. |
| | Camphor, - - - - - | gr. xv. |
| | | M. |

### Coryza of Adults and Children.

Tannin is regarded as a valuable remedy in this usually troublesome and intractable affliction. For adults it is snuffed into the nose in small pinches, three or four times a day, as in the following formula, viz:

| | | |
|---|---|---|
| ℞ | Tannin, - - - - - - | gr. ¾ |
| | Pulv. Iris, | |
| | " Althea, - - - - - | *aa* grs. 15. |
| | Tinct. Vanillæ, - - - - - | gtt. 4. |
| | | M. |

For *Children* the following formula is used by smearing it on small cylinders of paper and then introducing them deeply into each nasal fossa, two or three times a day, until sneezing occurs, viz:

| | | |
|---|---|---|
| ℞ | Tannin, - - - - - - | gr. ¾ |
| | Ung. Simp. - - - - - - - | ℥ i, ℈ ii. |
| | Tinct. Vanillæ - - - - - | qtt. v. |
| | | M. |

### Cancer.

*Acetic acid,* locally applied, is said to possess extraordinary virtues.

*Perchloride of Iron* is regarded by M. Bitol, of Bordeaux, as a *specific* for cancer when used both internally and externally.

### Epithelioma, (Cancer.)

*Chloride of Zinc Paste* and Sulphate of Zinc, as well as

Arsenic are all recommended as local applications in cancer. The former should be mixed well with *three* parts of wheaten flour. The internal administration of *Iron* and *Arsenic* should also be used.

CHOLERA. (Ethiop's Mineral.)

*Black Sulphuret of Mercury.*—Dr. Socrates Cadet, Professor at Rome, regards the use of ETHIOP'S MINERAL as *specific* in cholera. He advises it in doses of *four grains* several times a week as a preventative, and *twelve to thirty grains* several times repeated as a curative.

REID'S CHOLERA MIXTURE

Should be given as follows: As soon as bowel complaint makes its appearance, take a *teaspoonful* of the syrup and go to bed. Repeat the dose every time the bowels are moved, increasing it if the first proves insufficient. Children should be dosed in proportion to age.

Formula as follows.

| | | |
|---|---|---|
| ℞ | Chloroform, - - - - - | f ʒ i. |
| | Gum Camphor, pulv., - - - | ʒ iii. |
| | Tinct. opium, - - - - - | f. ℥ ij. |
| | Best French Brandy, - - - - | f. ℥ iv. |
| | Tinct. Cloves, - - - - - | f. ℥ i. |
| | Syruple syrup, - - - - - | f. ℥ viij. |
| | | M. |

OUR CHOLERA MIXTURE.

| | | |
|---|---|---|
| ℞ | Tinct. Capsicum, - - - - | f. ʒ iv. |
| | " Digitalis, - - - - - | f. ʒ ij. |
| | Chloroform, - - - - - | f. ʒ ii. |
| | Tincture Myrrh, - - - - | f. ʒ ij. |
| | " Nucis Vomica, - - | f. ʒ i. |
| | " Opium, - - - - | f. ʒ vi. |
| | Spts. Lavender Comp, - - - | f. ʒ iv. |
| | Aquæ Camph., - - - - - | f. ℥ iss. |
| | | M. |

S. One teaspoonful and a-half, in a little water, keeping the patient *in bed*, *requiring* him to use the bedpan.

H. L. Byrd, M. D.

OPIUM IN CHOLERA.

Dr. Brown Sequard recommends large doses of opium in the treatment of cholera. Laudanum is preferred in 20 drop doses every 15 or 20 minutes, until some slight signs of opium poisoning are produced. He recommends *actual cautery* to the back when the urinary secretion is suspended.

CORROSIVE SUBLIMATE in *chronic* and obscure NERVOUS DISEASES, when continued for some time, has been found wonderfully efficacious. As *Epilepsy*, partial *paralysis*, chronic *brain diseases*, etc. To be successful the Corrosive sublimate should be continued for weeks and even months.

CATARRH IN THE HEAD

Rub together in a mortar five grains *sulph. quinine* and twenty-five grains of white sugar, and use as snuff several times a day.

CONSUMPTION OR CHRONIC BRONCHITIS

| | | |
|---|---|---|
| ℞ | Fluid Ext. Ergot, . . . . | f. ʒ xss. |
| | Tinct. Aconite Root. | |
| | " Verat Vivid, . . . . . | *aa* M. 64 |
| | " Tinct. Digitalis, . . . | f. ʒ v. |
| | Lig. Pot. Arsenitis, . . . . . | M. 128. |
| | Syrup Totutan. | |
| | Glycerine, . . . . . | *.aa* ℥ iiss. |
| | | M. |

S. One teaspoonful three times a day in a little whiskey or Brandy.

COUGH OF CONSUMPTION.

| | | |
|---|---|---|
| ℞ | Potassii Bromid. | |
| | Potassæ Chloratis. | |
| | Ammonia Muriatis, . . . . . | *aa* ʒ iss. |
| | Syrup Tolutanis, . . . . . . . | ℥ iv. |
| | | M. |

S. A teaspoonfull every two or three hours.

COUGH *of any chronic character*, as bronchitis, etc.,

| | | |
|---|---|---|
| ℞ | Tinct opii camphoratæ, . . . . | ℥ i. |
| | " Belladonnæ, . . . . . . | ʒ i. |
| | " Hyoscyami, . . . . | ʒ ij. |
| | Spirits Lavendulæ campos, . . . | ʒ i. |
| | | M. |

S. Ten drops on a lump of sugar every hour until cough is relieved.

CHOLERA MORBUS.

| | | |
|---|---|---|
| ℞ | Pulv. Epecac, . . . . . . | gr. ½ |
| | " Opium, . . . . . | gr. ¼ to ½ |
| | " Cinnamon, . . . . | gr. ½. |
| | | M. |

This dose is administered every four hours, and as the symptoms subside, every eight hours. The Ipecac is advantageously increased until three grain doses are reached, with pellets of ice to allay thirst. Even cases where large quantities of blood are passed are often speedily relieved.

CHLOROFORM COUGH MIXTURE.—This is prepared as follows:

| | | |
|---|---|---|
| ℞ | Morph. acet, . . . . . . | gr. iij. |
| | Tinct. Belladonnæ, . . . . . | ʒ ij. |
| | Spts. chloroformi, . . . . . | ʒ vi. |
| | Syr. Senegæ, . . . . . | ℥ j. |
| | Syr. pruni virg. ad.,. . . . . | ℥ iv. |
| | | M. |

Dose, one teaspoonful three times per day.

CODEA *in painful Menstruation and Renal colic*, In ¼ grain doses. Dr. Oliver, of New Jersey, speaks of it also in exalted terms in Mani-a-potu.—*College and Clinical Record.*

